# Screening of compound-formulated *Bacillus* and its effect on plant growth promotion

**DOI:** 10.3389/fpls.2023.1174583

**Published:** 2023-05-10

**Authors:** Yuying Shen, Huan Yang, Zheng Lin, Linglong Chu, Xiao Pan, Yu Wang, Wenbo Liu, Pengfei Jin, Weiguo Miao

**Affiliations:** Key Laboratory of Green Prevention and Control of Tropical Plant Diseases and Pests, Ministry of Education and School of Plant Protection, Hainan University, Haikou, China

**Keywords:** *Bacillus*, plant growth promotion, eco-friendly, bacterial solution compounding, secondary metabolite

## Abstract

*Bacillus* bacteria can produce abundant secondary metabolites that are useful for biocontrol, especially in maintaining plant root microecology, and for plant protection. In this study, we determine the indicators of six *Bacillus* strains for colonization, promotion of plant growth, antimicrobial activity, and other aspects, with the aim of obtaining a compound bacteriological agent to construct a beneficial *Bacillus* microbial community in plant roots. We found that there was no significant difference in the growth curves of the six *Bacillus* strains over 12 h. However, strain HN-2 was found to have the strongest swimming ability and the highest bacteriostatic effect of n-butanol extract on the blight-causing bacteria *Xanthomonas oryzae* pv. *oryzicola*. The hemolytic circle produced by the n-butanol extract of strain FZB42 was the largest (8.67 ± 0.13 mm) and had the greatest bacteriostatic effect on the fungal pathogen *Colletotrichum gloeosporioides*, with a bacteriostatic circle diameter of 21.74 ± 0.40 mm. Strains HN-2 and FZB42 can rapidly form biofilms. Time-of-flight mass spectrometry and hemolytic plate tests showed that strains HN-2 and FZB42 may have significantly different activities because of their ability to produce large quantities of lipopeptides (i.e., surfactin, iturin, and fengycin). Different growth-promoting experiments revealed that the strains FZB42, HN-2, HAB-2, and HAB-5 had better growth-promoting potential than the control, and therefore these four strains were compounded in an equal ratio and used to treat pepper seedlings through root irrigation. We found an increase in the stem thickness (13%), leaf dry weight (14%), leaf number (26%), and chlorophyll content (41%) of pepper seedlings treated with the composite-formulated bacterial solution compared to the optimal single-bacterial solution treatment. Furthermore, several of these indicators increased by an average of 30% in the composite solution-treated pepper seedlings compared with the control water treatment group. In conclusion, the composite solution obtained by compounding strains FZB42 (OD_600_ = 1.2), HN-2 (OD_600_ = 0.9), HAB-2 (OD_600_ = 0.9), and HAB-5 (OD_600_ = 1.2) in equal parts highlights the advantages of a single bacterial solution, which includes achieving good growth promotion and antagonistic effects against pathogenic bacteria. The promotion of this compound-formulated *Bacillus* can reduce the application of chemical pesticides and fertilizers; promote plant growth and development; avoid the imbalances of soil microbial communities and thus reduce the risk of plant disease; and provide an experimental basis for the production and application of various types of biological control preparations in the future.

## 1 Introduction

Modern agricultural production inevitably relies on the application of chemical fertilizers and chemical pesticides to increase yields. However, excessive use can cause an imbalance of environmental nutrients in the soil, causing serious damage to the agricultural environment. This can lead to an annual decline in the yield and quality of agricultural products, threatening food security and causing irreversible damage to the ecological environment (Mosa et al., 2016). Therefore, there is an urgent need to develop microbial agents that are not harmful to the environment and that are safe and effective (Zvinavashe et al., 2019; Trivedi et al., 2020).

Microbial agents are a class of active compounds that provide protection against pathogenic bacteria. One of these microbial agents, rhizobia, can prevent soil-borne diseases and can positively affect rhizomes (Grundy et al., 2023). Compared with traditional pesticide and fertilizer applications, plant growth-promoting rhizobacteria (PGPR) strains are widely used because of their limited environmental harm, sustained improvements to soil fertility, and prevention of soil-borne diseases (Cao et al., 2012; Qiu et al., 2012). The most studied strains of PGPR are Gram-positive *Bacillus* bacteria, which can promote plant growth by producing lipopeptides (e.g., surfactin, iturin, and fengycin), synthesizing and secreting phytohormones to prevent pathogenic bacteria from attacking.

*Bacillus* is a widely distributed genus of Gram-positive bacteria with a rich diversity of physiological characteristics and is an important part of the microbial community in soil and plant roots. *Bacillus* is able to produce abundant antimicrobial substances with potential plant disease control and agronomic applications, such as the lipopeptide antibiotics surfactin, iturin, and fengycin, which are synthesized by the non-ribosomal pathway (Li and Liu, 2015; Abriouel et al., 2011). Surfactin is not only a highly active biosurfactant; it has a wide range of applications, exhibiting antiviral (Saggese et al., 2018), antifungal (Gao et al., 2003), and, to a lesser extent, antibacterial activity (Zeriouh et al., 2014). Iturin interferes with fungal cytoplasmic membrane function through the formation of vesicles and the aggregation of inner membrane particles, promoting the release of electrolytes and macromolecules and the degradation of phospholipids, leading to cell death (Xiao et al., 2021). Similarly, *Bacillus* can stimulate plant growth by synthesizing phytohormones such as indole-3-acetic acid (IAA), gibberellin, and cytokines. IAA is a growth hormone that acts throughout plant growth and development. IAA affects plant cell division, elongation, and differentiation, seed germination, root development, and nutritional growth processes (Phillips et al., 2011). In dicotyledonous plants, IAA promotes the production of lateral roots. In monocotyledonous plants, IAA promotes the formation of adventitious roots. IAA responds to light, gravity, and plant flowering and fruiting stages and is important for plant photosynthesis, pigment formation, the synthesis of various metabolites, and stress resistance (Woodward et al., 2010). In addition, *Bacillus* can control soil-borne pathogens through mechanisms such as antagonism, competition, induction of systemic resistance, and hyperparasitism (Siddiqui, 2005). After entering the inter-root environment, *Bacillus* promotes growth and controls disease by colonizing the root surface and interacting with microorganisms in the plant and surrounding soil (Ma et al., 2018).

In this study, we compared six strains of *Bacillus* for their biological properties, inhibition ability, and growth-promoting effect on *Capsicum chinense* pepper seedlings, and we found that each strain had unique biological characteristics. We screened the four strains with the most effective growth promotion and the strongest antimicrobial activity: FZB42 (OD_600_ = 1.2), HN-2 (OD_600_ = 0.9), HAB-2 (OD_600_ = 0.9), and HAB-5(OD_600_ = 1.2). Combined in a 1:1:1:1 ratio formula, the strains enhance each other’s strengths and show good colonization, growth promotion, and bacterial inhibition, providing a theoretical basis for further research into the production and application of the compounded preparations of various *Bacillus* biological agents.

## 2 Materials and methods

### 2.1 Bacterial and fungal strains and growth conditions

*Bacillus velezensis* HN-2 and *Bacillus amyloliquefaciens* HN-3 from a rice field in Hainan province and *Bacillus amyloliquefaciens* HAB-2 and *Bacillus subtilis* HAB-5 from the soil of a cotton field in Xinjiang province were isolated in the author’s laboratory, while *Bacillus amyloliquefaciens* FZB42 and *Bacillus subtilis* 168 were generously donated by Professor Gao of Nanjing Agricultural University. The *Colletotrichum gloeosporioides* strain BWZA was provided by the China Center for Type Culture Collection (ID. CCTCC AF 2015008) and cultured in potato dextrose agar and carboxymethylcellulose sodium medium at 28°C. *Xanthomonas oryzae* pv. *oryzicola* (*Xoo*) was cultured in a peptone sucrose agar (PSA) medium at 28°C. The bacterial organophospate medium consists of glucose 10 g, (NH_4_)_2_SO_4_ 0.5 g, yeast extract 0.5 g, NaCl 0.3 g, KCl 0.3 g, MgSO_4_ 0.3 g, FeSO_4_ 0.03 g, MnSO_4_ 0.03 g, lecithin 0.2 g, and CaCO_3_ 1.0 g, at 28°C. A Columbia blood agar plate, purchased from www.bio-caring.cn, and Cooperative 903 *Capsicum chinense* peppers from the Horticultural Research Institute, Shanghai, were used.

### 2.2 Determination of swarming motility and biofilm formation ability of six *Bacillus* strains

Six single colonies of *Bacillus* spp. were picked sequentially on Luria–Bertani (LB) plates, then inoculated in an LB liquid medium and incubated at 28°C at 180 rpm until the absorbance A_600nm_ reached 1.0. First, 5 μL of the bacterial suspension was carefully pipetted into the center of swarm plates prepared 1 day before use with 25 mL of LB medium fortified with 0.7% agar (10 g of tryptone, 5 g of yeast extract, and 5 g of NaCl per liter) (Patrick and Kearns, 2009; Salvetti et al., 2011). The plates were placed in an incubator at 28°C and observed every 2 h, and the radius of colony expansion was recorded until the entire plate was covered. Subsequently, 20 μL of bacterial suspension was mixed with 2 mL of minimal salt glycerol-glutamate medium and added to the 24-well cell culture plate, incubated at 28°C, and the formation of biofilms from six strains of *Bacillus* was observed (Yu, 2016). In an experiment, the swarm assays were performed in triplicates, and at least three independent experiments were performed.

### 2.3 Extraction of active substances by n-butanol extraction

The target strains were transferred into the LB medium in 250-mL Erlenmeyer flasks and subjected to a 48-h shaking incubation at 180 rpm at 28°C. The target strains were then centrifuged at 10,000 rpm for 10 min to remove the bacteria and obtain the fermentation supernatant. The fermentation supernatant was extracted with n-butanol organic solvent at a ratio of 1:1 to obtain the upper n-butanol extracts, and then freeze-dried after rotary evaporation to obtain the n-butanol crude extracts (Stein, 2005).

### 2.4 The hemolysis assay for surface activity

Under sterile conditions, the crude extracts from the n-butanol extraction were dissolved in methanol to a final concentration of 50 mg/mL, and 10 μL was added dropwise on a 6-mm diameter filter paper plate. After drying, the filter paper was placed symmetrically on the blood agar plate, blow-dried, and then placed upside down in a 28°C incubator. The diameter (mm) of the transparent circle was then measured with a vernier caliper after 12 h (Yu, 2016).

### 2.5 Effect of n-butanol crude extracts on *Xanthomonas oryzae* pv. *oryzicola*

The strain *Xoo* was transferred to a PSA medium in 250-mL Erlenmeyer flasks and subjected to 24-h shaking incubation at 180 rpm at 28°C until it reached an absorbance A_600nm_ of 0.3. Subsequently, 0.1 mL of the pathogenic liquid was evenly spread on the PSA nutrient plate medium. The crude extracts of n-butanol were dissolved in methanol to a final concentration of 1 mg/mL, and 10 μL was added dropwise on 6-mm diameter filter paper. After drying, the filter paper was placed symmetrically on the plate. After incubation for 48 h at 28°C, the diameter of the inhibition ring was measured with a vernier caliper (Wang et al., 2020).

### 2.6 Effect of n-butanol crude extracts on *Colletotrichum gloeosporioides*

The strain *Colletotrichum gloeosporioides* was transferred to a carboxymethylcellulose sodium medium in 250-mL Erlenmeyer flasks and subjected to 3 to 5 days of shaking incubation at 180 rpm at 28°C. The medium was filtered with sterile gauze to remove mycelia, resulting in a spore suspension that was mixed 1:1 with the potato dextrose agar medium. The crude n-butanol was dissolved in methanol to a final concentration of 50 mg/mL and pipetted (10-μL) onto filter paper with a diameter of 6 mm. Sterile water was used as a negative control. After drying, the filter paper was placed symmetrically on the plate and incubated at 28°C for 48 h. The diameter (mm) of the inhibition ring was measured with vernier calipers (Jin et al., 2020; Wang et al., 2020).

### 2.7 Chemical analysis of lipopeptides from *Bacillus* spp.

Matrix-assisted laser desorptionionization–time of flight (MALDI-TOF) mass spectrometry was employed to analyze lipopeptides in *Bacillus* spp. The culture filtrate of *Bacillus* spp. was acidified to pH 2 with 6 N HCl solution and stored at 4°C overnight, and then centrifuged. The precipitate was dissolved in methanol and filtered through a 0.2-μm polytetrafluoroethylene membrane. The experiment was performed on a Bruker Daltonics Reflex MALDI-TOF mass spectrometer with a Scout-mtp ion source containing a 337-nm nitrogen laser. All spectra were acquired in the reflector positive ion mode. The acceleration and reflector voltages were 25 kV and 26.3 kV, respectively. The matrix medium was a saturated solution of α-cyano-4-hydroxycinnamic acid in 30% aqueous acetonitrile containing 0.1% trifluoroacetic acid (v/v). For MALDI-TOF analysis, a 1-μL extract was added to the target plate with 1 μL of working solution and then air-dried (Jin et al., 2017).

### 2.8 Qualitative determination of the ability to secrete plant growth factor (IAA)

Salkowski’s colorimetric solution (Glickmann and Dessaux, 1995) was prepared by weighing 12 g of FeCl_3_, dissolving it in 300 mL ddH_2_O, and slowly adding 429.7 mL of 98% H_2_SO_4_. This was then allowed to cool down to a fixed volume of 1 L. The ability of six *Bacillus* strains to secrete IAA was determined by the Salkowski colorimetric method. Six strains of *Bacillus* were inoculated and cultured in an LB liquid medium, with shaking for 24 h (28°C, 180 rpm); 1 ml of the bacterial fluid was then aspirated and centrifuged at 5,000 rpm for 10 min, and the supernatant was discarded. After washing the bacteria twice with ddH_2_O and adjusting the bacterial suspension to an OD_600_ = 0.05, 100 μL of the bacterial suspension was inoculated into 50 mL of a King B medium containing 100 mg/L of L-trip. Subsequently, 100 μL of sterile water was added for CK, placed in a 28°C shaker at 180 rpm, and shaken for 24 h. Six *Bacillus* spp. bacteria suspensions and CK were pipetted. After centrifugation at 10,000 rpm for 10 min, 2 mL of the supernatant was added to the EP tube, protected from light, mixed with 2 mL of Salkowski’s reagent, and allowed to stand for 30 min at room temperature, again protected from light, and the absorbance value at A_530nm_ was determined. The IAA production assays were performed in triplicate, and at least three independent experiments were performed.

### 2.9 Determination of dissolved organic phosphorus content of six strains of *Bacillus* spp.

The six strains of *Bacillus* spp. were spotted on the bacterial organophosphorus medium with sterile toothpicks and incubated upside down in an incubator at 28°C. The size of the transparent circle was observed and recorded after 7 days. The six strains of *Bacillus* spp. were transferred to 100 mL of a bacterial organophosphorus medium at 28°C. After 7 days of shaking the culture, 2 mL of the fermentation solution was aspirated, it was centrifuged at 12,000 rpm for 10 min, the supernatant was aspirated, and ddH_2_O was added until at 30 mL. The solution was adjusted with 6 mol/L of NaOH solution to achieve a slightly yellow color, and 5 mL of molybdenum antimony anti-reagent was added. (5 g of ammonium molybdate was dissolved in 225 mL of ddH_2_O, 76.5 mL of H_2_SO_4_ was added slowly and stirred, 100 mL of a 5 g/L potassium antimony tartrate solution was added when the liquid temperature dropped to room temperature, and the volume was fixed with a 500-mL ddH_2_O volumetric flask. The solution was shaken and placed in a brown bottle. Before use, 1.5 g/L of ascorbic acid was added to each 100 mL of molybdenum-antimony anti-reagent.) The absorbance value at A_700nm_ was measured after standing for 30 min at room temperature, and the soluble organic phosphorus content in the fermentation broth was calculated (Li, 2020). The dissolved organic phosphorus content assays were performed in triplicates, and at least three independent experiments were performed.

### 2.10 Detection of a growth-promoting effect on pepper

We used 75% ethanol for 2 min (with constant shaking during this time), added 2% NaClO to cover the seeds, used a shaker to shake at 160–170 rpm for 5–7 min to achieve surface disinfection, and then, they were soaked in the broth of six strains of *Bacillus* for 24 h, in which the broth of each Bacillus contained five concentration treatments (OD600 = 0.3, 0.5, 0.7, 0.9, and 1.2. At an OD600 = 1 cell count concentration of 2 × 10^9^ CFU/mL. A total of 200 seeds were treated for each concentration.). The seeds were sown in 0.7% agar medium, and the germination of the seeds was observed and recorded after 10 days of incubation. After seed germination, the seeds were cultured in a greenhouse at a constant temperature of 26°C under alternating 16 h of light and 8 h of darkness, and the stem length and root length of pepper seedlings were measured and recorded for each treatment after 20 days. The figures were statistically analyzed using SPSS statistical software (three replicates for each treatment).

### 2.11 Experiment on the growth-promoting effect of four strains of *Bacillus* and their compound formulation on pepper

For pepper seed cultivation, strains FZB42, HN-2, HAB-2, and HAB-5 were inoculated, with the greatest pro-growth effect determined in the above experiments, into an LB medium in 250-mL Erlenmeyer flasks and subjected to a 24-h shaking incubation at 180 rpm at 28°C, and then centrifuged (8,000 rpm, 5 min). The bacterial body was washed twice with ddH_2_O, and the bacterial suspension absorbance was optimally adjusted to A_600nm_. Finally, we took an equal amount of bacteria suspension (i.e., the same proportion of the suspension used to form a compound solution) from four strains of *Bacillus*. The pepper seeds were soaked in the above five bacterial suspensions overnight in groups of 10 and then placed in plastic pots with 1:1 vermiculite and nutrient soil, and the seeds were covered with a thin layer of soil and watered regularly.

For the addition of the strain fermentation solution, after the seeds of the peppers broke through the soil and germinated, 200 mL (concentration 10^6^ CFU/mL) of fermentation solution was poured into the roots of pepper seedlings in each pot, and the blank group was filled with the same amount of ddH_2_O. The root length, stem thickness, dry weight, and chlorophyll content of the peppers were measured after 40 days.

### 2.12 Determination of chlorophyll content

We took 0.3 g of fresh pepper leaves, wiped the tissue surface dirt off, removed the mid-vein, and cut. Then, we put them into a mortar, added a small amount of calcium carbonate and quartz sand, and 3 mL of anhydrous ethanol, ground this into a homogenous slurry, added 10 mL of anhydrous ethanol, continued to grind, and left it to stand for 10 min. We then moistened it with anhydrous ethanol, poured the extract into a funnel lined with filter paper along the glass rod, filtered it into a 25-mL brown volumetric flask, rinsed the mortar, grinding rod, and residue several times with a small amount of anhydrous ethanol until there was no green color in the filter paper and residue, fixed the volume to 25 mL with anhydrous ethanol, and, finally, shook well. The chlorophyll extract was poured into a 1-cm-diameter light colorimetric cup, and the absorbance was measured at OD_649_ and OD_665_ with anhydrous ethanol as a blank control. The values were substituted into the equation to find chlorophyll a (C_a_), chlorophyll b (C_b_), and total chlorophyll (C_T_). The chlorophyll content assays were performed in triplicate, and at least three independent experiments were performed.


$$C=\frac{CT\times V\times 1}{W}$$

$$C_{T}-C_{a}+C_{b}\;C_{\alpha }-13.95OD_{665}-6.88OD_{649}$$

$$C_{b}-24.96\;OD_{649}-7.32\;OD_{665}\;V-Extraction\;solution\;volume,\;mL$$

W−Sample fresh weight, g      C−Chlorophyll content, mg/g·FW

### 2.13 Data analysis

Experimental data were analyzed using a one-way ANOVA and SPSS 2.5 software and presented as mean values of the treatment replicates ± standard deviation. For multiple comparisons of means and evaluation of significant differences between treatments, Duncan (DC) was used at *p* = 0.05. Values are expressed as mean ± standard deviation. Experiments were conducted three times, with three replicates each. One asterisk (*) indicates a significant difference (*p*< 0.05), two asterisks (**) indicate a highly significant difference (*p*< 0.01), and three asterisks (***) indicate a significant difference (*p*< 0.001) between the two groups with significant differences by the DC test.

## 3 Results

### 3.1 Determination of swarming motility and biofilm formation ability of six *Bacillus* strains

**Figure 1:**
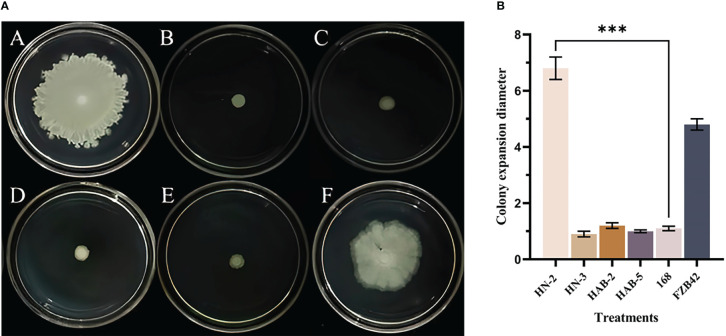
The swarming motility assay of six species of *Bacillus*. **(A)**: The swarming motility assay of six species of *Bacillus* (morphological observation) A: strain HN-2; B: strain HN-3; C: strain HAB-2; D: strain HAB-5; E: strain 168; F: strain FZB42). **(B)**: The swarming motility assay of six species of *Bacillus* (data analysis). (***) indicate a significant difference (P <0.001) between the two groups with significant differences by the DC test.

In the swarming motility experiment (**Figure 1**), the result found that the swarming motility ability of strain HN-2 was the strongest, with a diffusion diameter of 60.74 ± 0.30 mm; the strain FZB42 was the second strongest, with a diffusion diameter of 52.18 ± 0.30 mm. The fastest expansion rate of strain HN-2 was clearly observed, and the colony that grew out formed a thin circle and was almost milky white at the beginning. The bacteria showed continued growth. After about 24 h, the colony that expanded was large and obvious. Among the remaining four strains of *Bacillus* (strains HN-3, HAB-2, HAB-5, and 168), there was no obvious sign of outward expansion; only a small circular colony with increasing thickness formed at the inoculation site.

**Figure 2:**
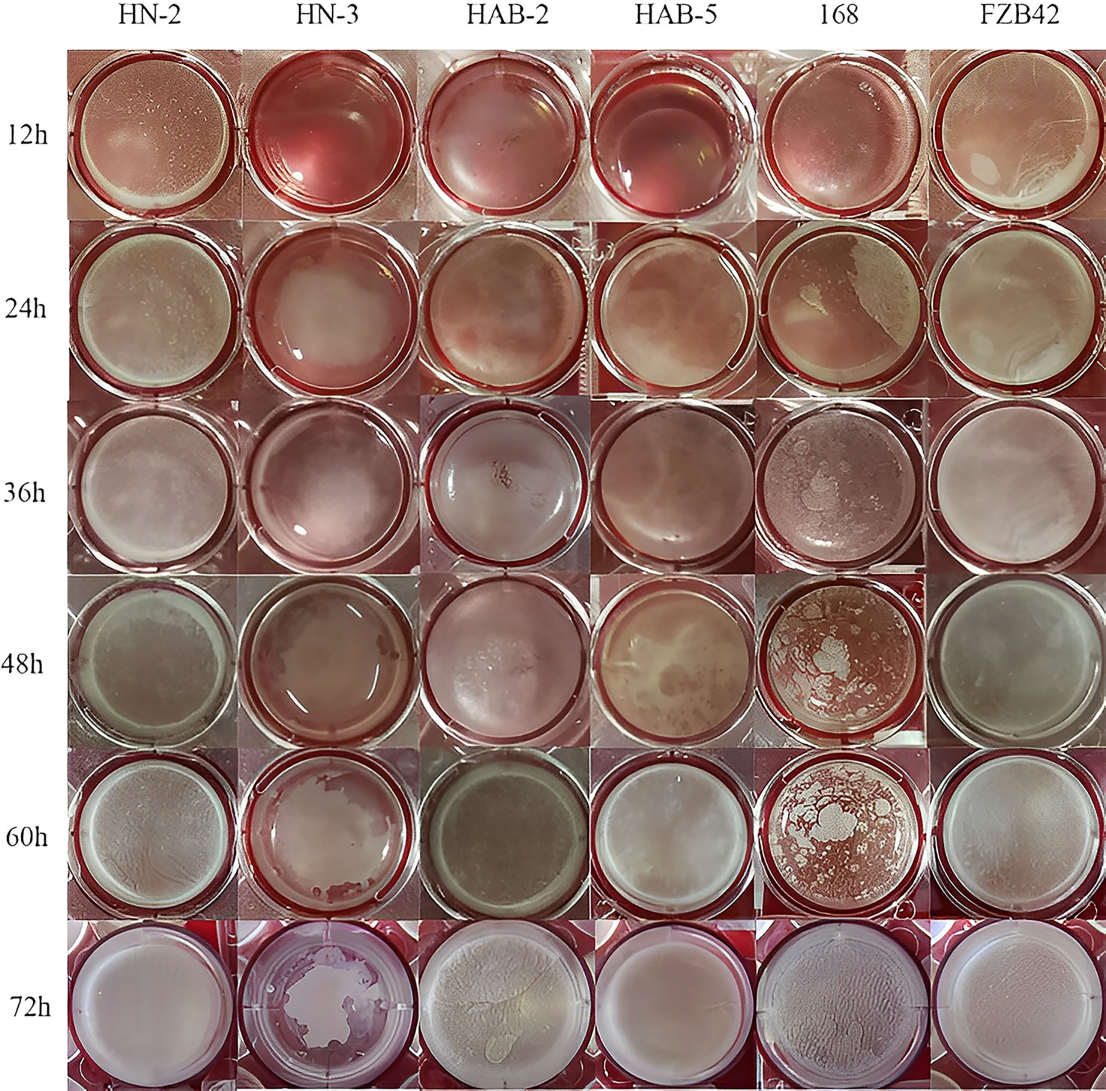
The biofilm phenotype of six species of *Bacillus*.

In a biofilm detection experiment (**Figure 2**), the biofilm formation ability of six strains of *Bacillus* species on the surface of a minimal salt glycerol-glutamate medium was qualitatively compared using a biofilm assay. The results showed that after 4 h of incubation, strains HN-2 and FZB42 started to form a biofilm; however, there were no significant changes in the remaining strains. As the incubation time increased, the biofilm of the other strains started to form and gradually thickened, showing obvious folds.

### 3.2 The hemolysis test to detect surface activity

**Figure 3:**
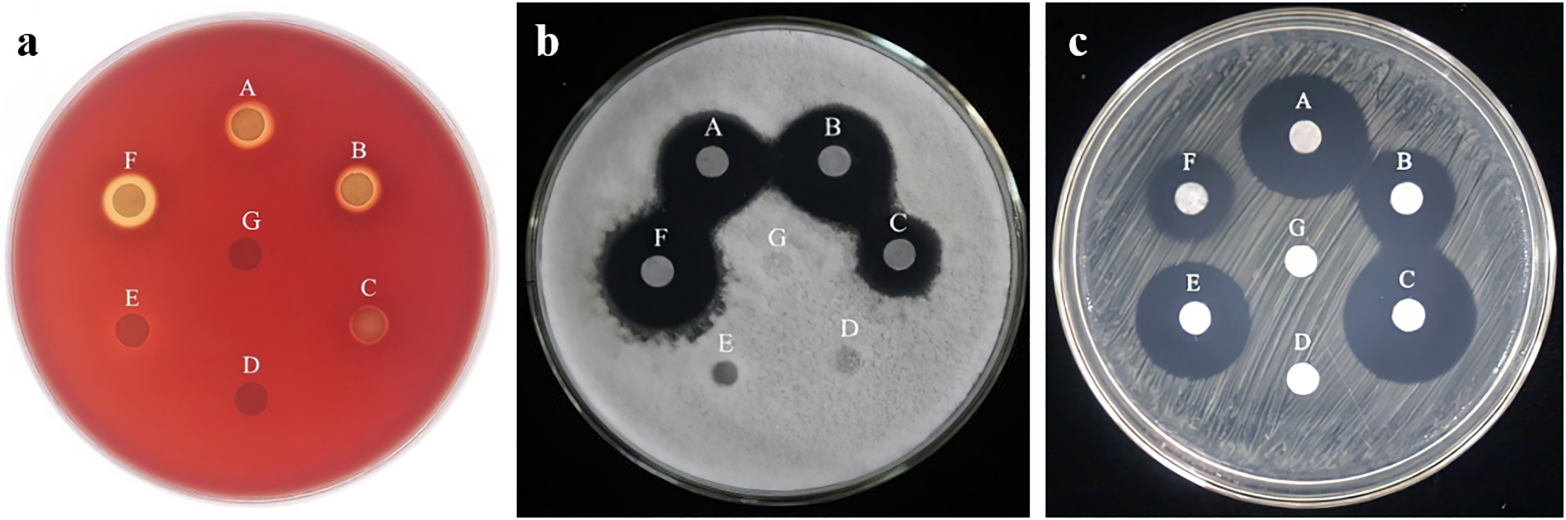
**(A)**: The hemolysis assay of six species of *Bacillus*. **(B)**: Inhibitory ability of n-butanol extracts of six strains of *Bacillus* spp. against *Colletotrichum gloeosporioides Penz* A: strain HN-2; B: strain HN-3; C: strain HAB-2; D: strain HAB-5; E: strain 168; F: strain FZB42; G: methanol). **(C)**: Inhibitory ability of n-butanol extracts of six strains of *Bacillus* spp. against *Xanthomonas oryzae* pv. *Oryzicola*A: strain HN-2; B: strain HN-3; C: strain HAB-2; D: strain 168; E: strain FZB42; F: strain HAB-5; G: methanol).

The surface activity of n-butanol-extracted lipopeptides was tested by blood plating (**Figure 3A**). The results showed that the lipopeptide compounds produced by strains HN-2, HAB-2, and FZB42 had an obvious hemolytic ability, with hemolytic circle diameters reaching 9.650 ± 0.01 mm, 10.32 ± 0.01 mm, and 10.72 ± 0.01 mm, among which FZB42 had the largest hemolytic circle diameter and the strongest hemolytic ability. The lipopeptide compounds produced by strains HN-3, 168, and HAB-5 were essentially non-hemolytic.

### 3.3 Effect of n-butanol crude extracts on *Colletotrichum gloeosporioides*

The antifungal activity of n-butanol extracts (50 mg/mL) of six strains of *Bacillus* was tested by a plate standoff experiment (**Figure 3B**). The n-butanol extracts of strains FZB42, HN-2, HN-3, and HAB-2 were found to have significant inhibitory effects, with inhibition ring diameters reaching 21.74 ± 0.05 mm, 20.07 ± 0.03 mm, 21.23 ± 0.30 mm, and 15.03 ± 0.33 mm, respectively. The n-butanol extracts of strain FZB42 lipopeptide had the best inhibitory activity, whereas strains 168 and HAB-5 had no antagonistic ability against fungi.

### 3.4 Effect of n-butanol crude extracts on *Xanthomonas oryzae* pv*. Oryzicola*

Using n-butanol extracts of lipopeptide compounds (1 mg/mL) inhibits *Xoo* (**Figure 3C**). The results showed that the diameters of the inhibition circles of strains HN-2, HN-3, HAB-2, and FZB42 were, respectively, 21.43 ± 0.08 mm, 17.46 ± 0.19 mm, 21.15 ± 0.02 mm, and 20.28 ± 0.16 mm. The HN-2 lipopeptides had the best inhibitory activity, whereas strains 168 and HAB-5 had no bacterial antagonistic activity.

### 3.5 Identification of crude extracts

The active substances extracted from the six strains of *Bacillus* were identified. Compared with the molecular weights based on time-of-flight mass spectrometry, the results showed that the active substances in the extract were mainly lipopeptides. The results indicated that the main active components of the n-butanol extract of strain HN-2 were surfactin A, B, and C, iturin A and B, bacillomycin D and F, and fengycin A and B; for strain HAB-2, they were iturin A, mycosubtilin, and bacillomycin F and L; for strain FZB42, they were bacillomycin L and iturin A and B; and for strain HAB-5, they were bacillomycin L and D (**Table 1**).

**Table 1:** Identification of lipopeptide species.

Strain	Ion-to-mass charge ratio	Compound
HN-2	1058.625	C_15_ Iturin B
		C_15_ Surfactin C
		C_16_ Bacillomycin D
		C_16_ Surfactin B
		C_15_ Surfactin A
	1121.815	C_17_ Bacillomycin F
		C_18_ Iturin A
	1066.660	C_14_ Iturin B
	1519.176	C_18_ Fengycin B
	1148.276	C_15_ Fengycin A
	1086.646	C_17_ Iturin B
HAB-2	1070.793	C_16_ Iturin A
		C_16_ Mycosubtilin
		C_15_ Bacillomycin F
	1062.451	C_17_ Bacillomycin L
HAB-5	1073.918	C_15_ Bacillomycin L
		C_17_ Bacillomycin D
	1034.167	C_15_ Bacillomycin L
	1053.110	C_14_ Bacillomycin D
FZB42	1020.685	C_14_ Bacillomycin L
	1043.503	C_14_ Iturin B
	1029.575	C_13_ Iturin A

### 3.6 Determination of the IAA content of six strains of *Bacillus*

**Figure 4:**
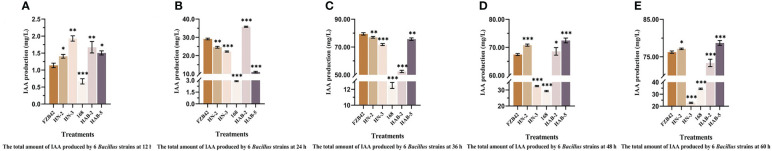
The total amount of indole-3-acetic acid (IAA) produced by six *Bacillus* strains at different times. **(A)**: 12 h. **(B)**: 24 h. **(C)**: 36 h. **(D)**: 48 h. **(E)**: 60 h. The experiments were conducted three times, each with three replicates. Experimental data were analyzed through one-way analysis of variance (ANOVA). One asterisk (*) indicates a significant difference (*p*< 0.05), two asterisks (**) indicate a highly significant difference (*p*< 0.01), and three asterisks (***) indicate a significant difference (*p*< 0.001) between the two groups with significant differences by the Duncan test.

The IAA production of the six *Bacillus* strains varied at different periods at the same inoculum level. In **Figure 4**, it can be seen that the IAA production of the six *Bacillus* strains would increase substantially at 36 h compared to 24 h, and the IAA production would be relatively flat at 48 h. Meanwhile, the IAA production of strains FZB42, HN-2, HAB-2, and HAB-5 was significantly higher than that of strains HN-3 and 168. The highest IAA yield of strain HN-2 at 60 h was 77.12 mg/L.

### 3.7 Determination of dissolved organic phosphorus content of six strains of *Bacillus*

**Figure 5:**
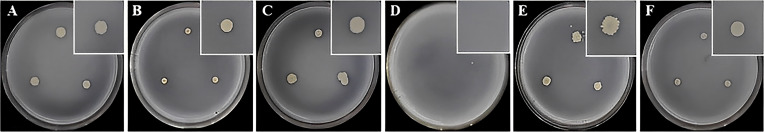
Phosphorus lytic efficacy of six strains of *Bacillus* spp. **(A)**: strain FZB42. **(B)**: strain HN-2. **(C)**: strain HN-3. **(D)**: strain 168. **(E)**: strain HAB-2. **(F)**: strain HAB-5.

**Figure 6:**
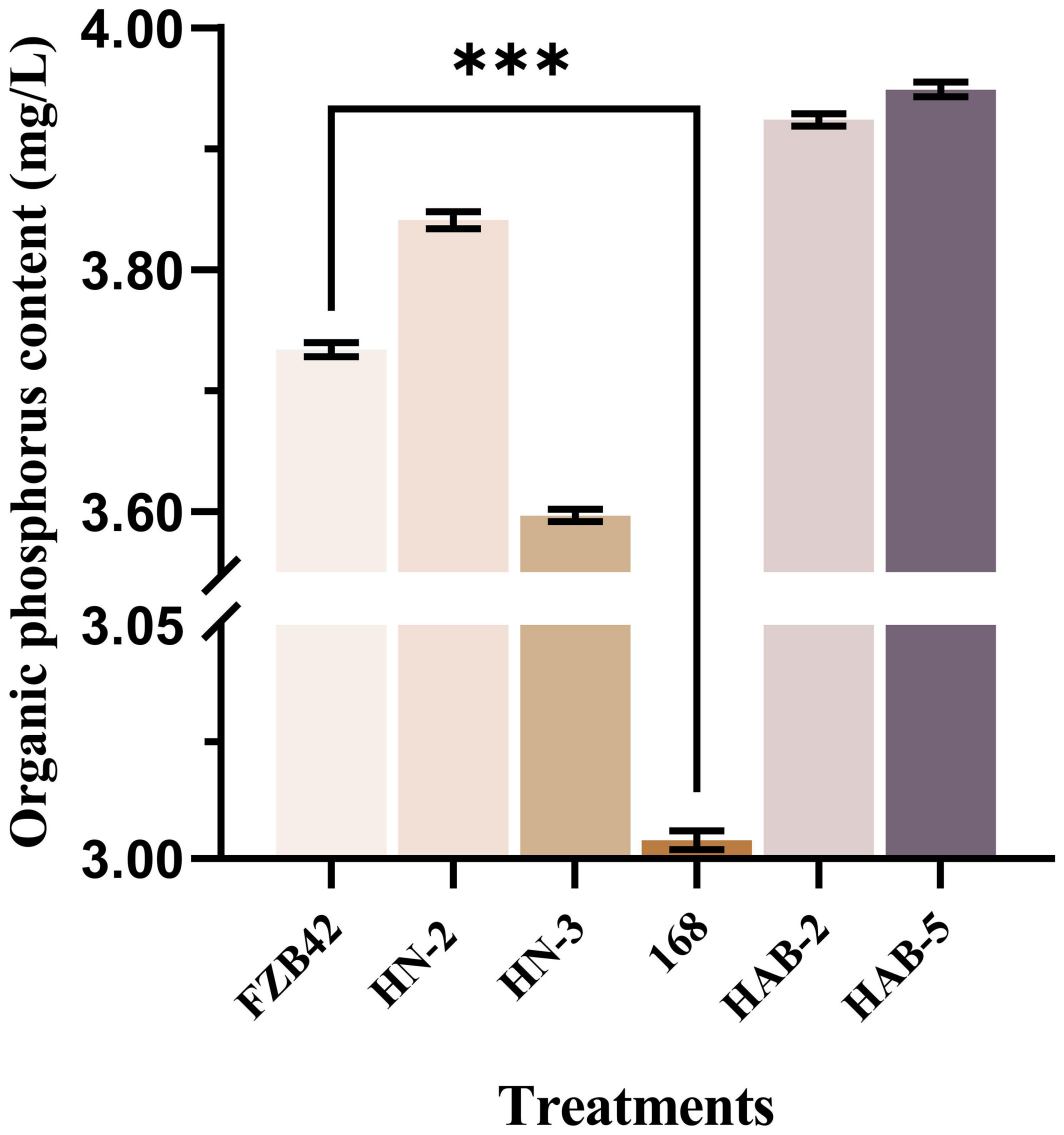
Organophosphorus content of *Bacillus* fluid after 7 days. The experiments were conducted. Three times, each with three replicates. A one-way ANOVA was used to compare the dissolved organic phosphorus content of six *Bacillus* strains after shaking for 7 days. Experimental data were analyzed through a one-way ANOVA. Asterisks (***) indicate a significant difference (P <0.001) between the two groups with significant differences by the Duncan test.

The six *Bacillus* strains were inoculated on an organophosphate bacterial medium for 7 days and clear circles were observed. It was initially determined that all strains except strain 168 had the ability to dissolve phosphorus (**Figure 5**). After the same inoculum amount of the six strains of *Bacillus* had been inoculated in organophosphate bacterial medium and shaken for 7 days, we tested the content of dissolved organic phosphorus in the supernatant and found that, except for strain 168, all *Bacillus* species obviously had the ability to dissolve organophosphorus. Among them, the amount of organic phosphorus that could be solubilized by strains FZB42, HN-2, HAB-2, and HAB-5 was more significant and reached a maximum of 3.949 mg/L (**Figure 6**).

### 3.8 Growth promotion assay on the root surface of pepper

**Figure 7:**
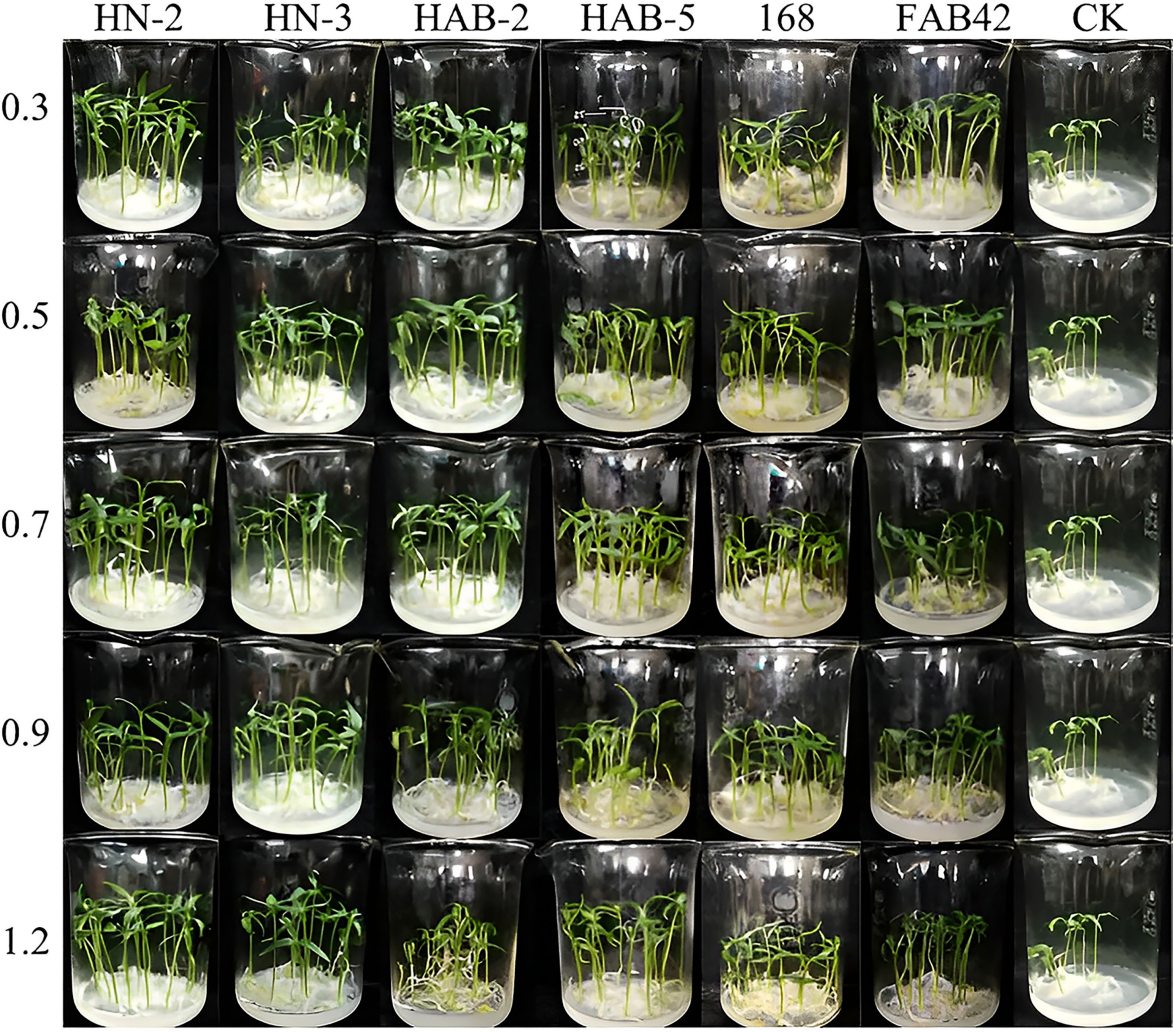
The growth promotion assay of six species of *Bacillus* on pepper. CK: no treatment control group was applied with sterile water; 0.3: the bacteria solution of OD_600_ = 0.3; 0.5: the bacteria solution of OD_600_ = 0.5; 0.7: the bacteria solution of OD_600_ = 0.7; 0.9: the bacteria solution of OD_600_ = 0.9; 1.2: the bacteria solution of OD_600_ = 1.2.

**Figure 8:**
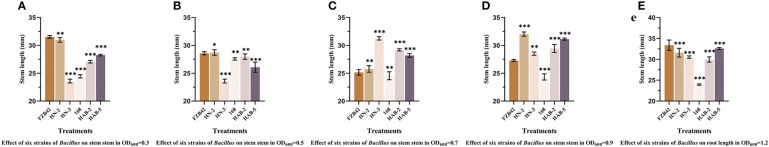
The detection of stem length in six strains of *Bacillus* on pepper promotion. **(A)**: OD_600_ = 0.3. **(B)**: OD_600_ = 0.5. **(C)**: OD_600_ = 0.7. **(D)**: OD_600_ = 0.9. **(E)**: OD_600_ = 1.2. The experiments were conducted three times, each with three replicates. Experimental data were analyzed through a one-way ANOVA. One asterisk (*) indicates a significant difference (*p*< 0.05), two asterisks (**) indicate a highly significant difference (*p*< 0.01), and three asterisks (***) indicate a significant difference (*p*< 0.001) between the two groups with significant differences by the Duncan test.

**Figure 9:**
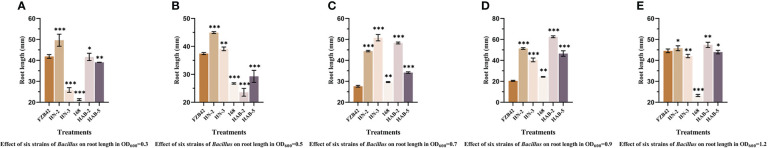
The detection of root length in six strains of *Bacillus* on pepper promotion. **(A)**: OD_600_ = 0.3. **(B)**: OD_600_ = 0.5. **(C)**: OD_600_ = 0.7. **(D)**: OD_600_ = 0.9. **(E)**: OD_600_ = 1.2. The experiments were conducted three times, each with three replicates. Experimental data were analyzed through a one-way ANOVA. One asterisk (*) indicates a significant difference (*p*< 0.05), two asterisks (**) indicate a highly significant difference (*p*< 0.01), and three asterisks (***) indicate a significant difference (*p*< 0.001) between the two groups with significant differences by the Duncan test.

In a pot culture of nutrient soil without other microbial community contamination (**Figures 7**–**9**), the growth promotion levels of five different bacterial solution concentrations of six *Bacillus* species showed that treatment with different bacterial solution concentrations of six *Bacillus* species (different bacterial solution concentrations were indicated by OD_600_) could promote the germination of pepper seeds and increase the germination rate, with the highest germination rate of pepper seeds treated with HN-2 bacterial solution (OD_600_ of 0.9) for 2 h reaching 100%. The germination rates of pepper seeds treated with FZB42 bacterial solution, HAB-5 bacterial solution (OD_600_ of 1.2), and HAB-2 bacterial solution (OD_600_ of 0.9) were 96%, 98%, and 90%, respectively, while the germination rate of pepper seeds treated with water was only 72%. Meanwhile, treatment with the bacterial solution of six strains of *Bacillus* showed a significant growth-promoting ability in both the stem and root tip of peppers. During the growth of pepper stems, the growth promotion effect of strain HN-2 was more stable in each concentration gradient; there was no significant difference between each concentration gradient, and it improved by about 14% compared with other strains. The overall growth promotion effect was the best. Strain FZB42 also showed an obvious growth promotion effect, but when the absorbanceA_600nm_ of the bacterial solution was 0.7, the growth promotion effect was significantly weakened, and the overall promotion effect showed the phenomenon of gradually weakening and then strengthening with the increase of absorbance A_600nm_. When the absorbance A_600nm_ of the bacterial solution was 1.2, the promotion effect of strain FZB42 was significantly higher than that of the other treatments by about 16%, and the corresponding stem length of the pepper reached 33.37 ± 0.03mm. When the absorbance A_600nm_ of the bacterial solution was 0.9–1.2, the promotion ability of strain 168 decreased with the increase of bacterial liquid concentration. The colonization promotion effect of strain HAB-2 increased with the increase in absorbance A_600nm_.

In terms of root growth, the promotion effect of HN-2 was stable across all concentration gradients. When the absorbance A_600nm_ of the bacterial solution was 0.9, the promotion effect of HAB-2 was significantly higher than the other treatments by about 32%, and the corresponding root length was 62.51 ± 0.03 mm. Before the absorbance A_600nm_ of the bacterial solution was 0.9, the root length of FZB42 became shorter as the absorbance A_600nm_ of the bacterial solution increased, and the most significant promotion effect was observed when the absorbance A_600nm_ was 1.2, with the root length reaching 44.54 ± 0.03 mm. However, the colonization and growth promotion abilities of each strain did not show a certain pattern with the increase in the absorbance A_600nm_ of the bacterial solution. The promotion effect on roots in the colonization experiment was significantly better than that on stems, and this may be because the biocontrol bacteria colonized the roots and the direct promotion effect on roots was greater.

Based on the data from the above experiments, we selected the four *Bacillus* spp. strains of (FZB42, HN-2, HAB-2, and HAB-5) that had the best inhibitory effect on bacteria and fungi and the best growth promotion effect on pepper seedlings for a 1:1:1:1 composite formulation according to the absorbance A_600nm_ of 1.2, 0.9, 0.9, and 1.2 and the obtained composite solution was used for subsequent experiments.

### 3.9 Experiment on the growth-promoting effect of four strains of *Bacillus* spp. and their compound formulation on pepper

**Figure 10:**
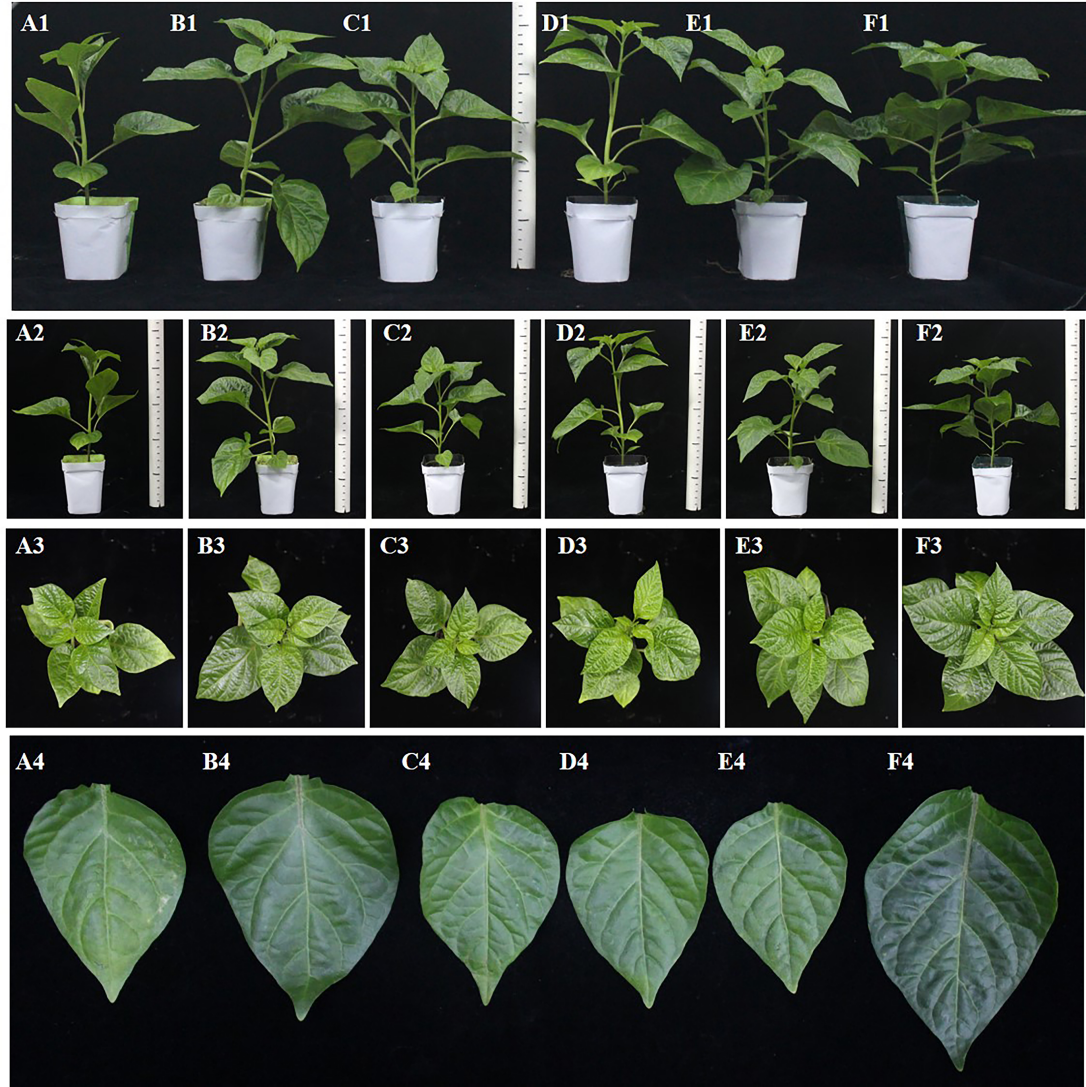
Growth promotion test of four strains of *Bacillus* and their complex solutions on pepper leaves. **(A1–A4)**: CK. **(B1–B4)**: FZB42. **(C1–C4)**: HN-2. **(D1–D4)**: HAB-2. **(E1–E4)**: HAB-5. **(F1–F4)**: compounding (**(A1–F1)** are the overall effect pictures; **(A2–F2)** are the effect plots of different treatments on plant height in pepper seedlings; **(A3–F3)** are plots of the effect of different treatments on leaf numbers in pepper seedlings; and **(A4–F4)** are the effect maps of different treatments on leaf size in pepper seedlings).

**Figure 11:**
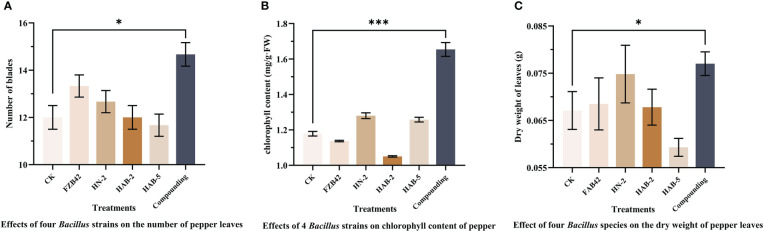
Proliferation of four strains of *Bacillus* and their complexes with respect to pepper leaves. The experiments were conducted three times, each with three replicates. One asterisk (*) indicates a significant difference (*p*< 0.05), and three asterisks (***) indicate a highly significant difference (*p*< 0.001) between strain FZB42 treatment and compound formulation treatment by the one-way ANOVA. **(A)** Determination of leaf number. **(B)** Determination of chlorophyll content. **(C)** Determination of leaf dry weight.

The total root length, stem diameter, chlorophyll content, number of leaves, and dry weight of pepper plants taken out of pots for 40 days were recorded. As shown in **Figures 10**, **11**, the number of leaves of peppers treated with the complex bacterial solution was significantly larger than that of peppers treated with the single bacterial solution by about 25%, reaching about 15 leaves per plant, followed by about 13 leaves treated with the FZB42 strain. After drying the same fresh weight of leaves, the dry weight of the pepper leaves treated with the complex bacterial solution was 0.077 g, which was 18% higher than that in the other four treatments. When the chlorophyll content of the leaves was measured, the chlorophyll content of the pepper leaves treated with the complex bacterial solution was found to be 1.654 mg/g, which was 58% higher than that of the pepper leaves treated with the single bacterial solution.

**Figure 12:**
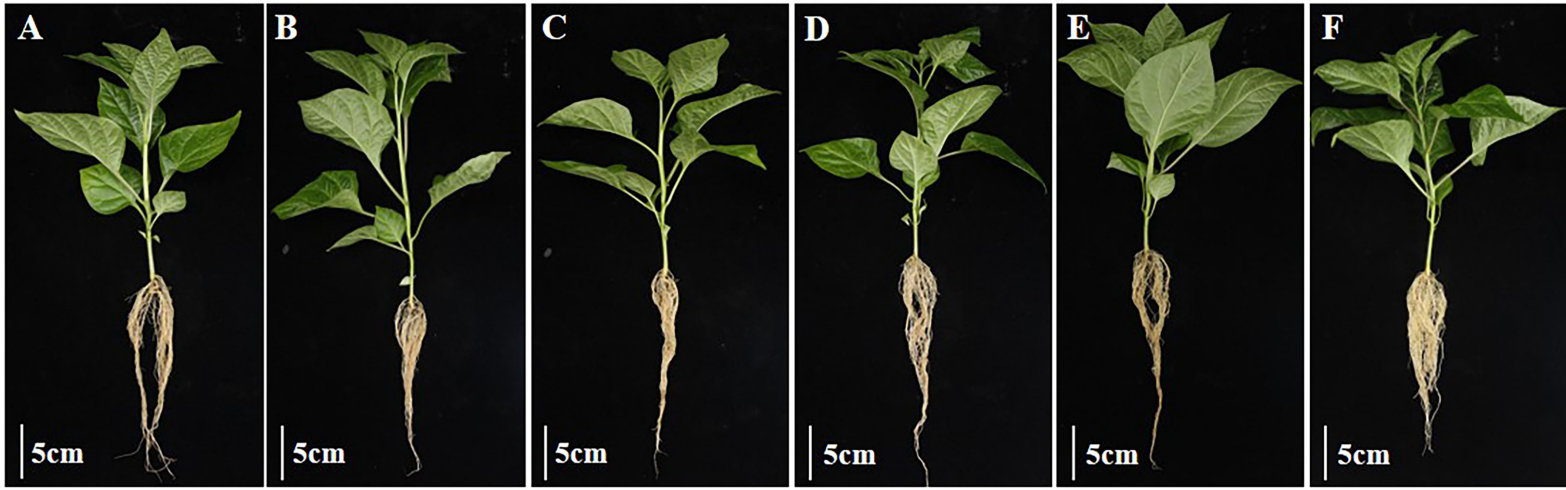
Growth promotion test of four strains of *Bacillus* and their complex solution on the rootstock of pepper. **(A)**: CK. **(B)**: FZB42. **(C)**: HN-2. **(D)**: HAB-2. **(E)**: HAB-5. **(F)**: compounding.

**Figure 13:**
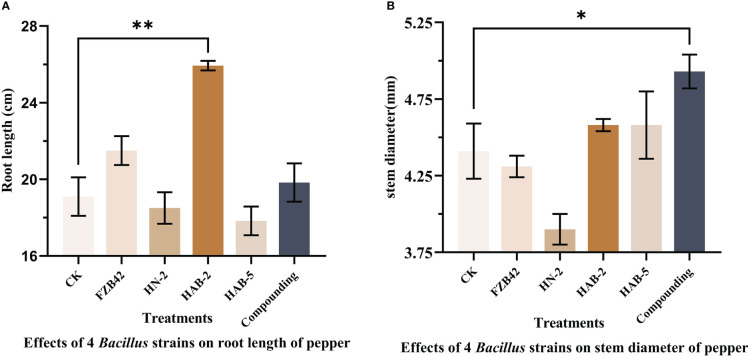
Growth promotion of pepper rhizomes by four strains of *Bacillus* and their complex solutions. The experiments were conducted three times, each with three replicates. One asterisk (*) indicates a significant difference (*p*< 0.05) between strain FZB42 and compound formulation treatment, and two asterisks (**) indicate a highly significant difference (*p*< 0.01) between strain FZB42 treatment and strain HAB-2 treatment by the one-way ANOVA. **(A)** Determination of root length. **(B)** Determination of stem diameter.

When measuring the total root length, it was found that the total root length of peppers treated with the complex bacterial solution was 19.83 cm, which was 4% longer than that of peppers treated with water. In terms of stem diameter, the pepper treated with the complex bacterial solution reached a stem diameter of 4.93 mm, which was 13% greater than that of the other four treatments and the water treatment. The pepper seedlings treated with the composite bacterial solution had the same excellent traits in all aspects as the four single bacterial solution treatments, indicating that there was no obvious competition between the four strains of *Bacillus* spp. selected for the composite formulation and that the composite solution could inherit the advantages of the four strains of *Bacillus* spp. for the growth promotion of pepper seedlings in all aspects very smoothly (**Figures 12**, **13**).

## 4 Discussion

*Bacillus*, as the first identified PGPR bacterium, plays an important role in plant roots as a growth-regulating agent (Éva et al., 2012; Hashem et al., 2019; Lugtenberg and Kamilova, 2009). Previous research has shown that *Bacillus* has significant effects on both germination and disease prevention, which improves plant growth. Furthermore, this bacterium secretes secondary metabolites, such as lipopeptides, which have inhibitory effects on pathogenic bacteria such as the fungal plant pathogen *Verticillium dahlie* and other *Bacillus* spp. (Wang, 2018; Yao, 2019). The biocontrol mechanisms of *Bacillus* include the secretion of bacteriostatic substances, promotion of plant growth, induction of plant systemic resistance, and competition for ecological niches in plant roots (Chen and Yu, 2020). In this study, we conducted phenotypic experiments to investigate the probiotic inhibitory effect of *Bacillus*, compared the different performances of the same phenotype of six different strains, and combined the six strains to determine their probiotic effect on the germination and growth of pepper seeds and their inhibitory effect on plant pathogenic bacteria, rice leaf blight, and blossom anthracnose. We used the best growth-promoting strains [FZB42 (OD_600_ of 1.2), HN-2 (OD_600_ of 0.9), HAB-2 (OD_600_ of 0.9), and HAB-5 (OD_600_ of 1.2)] in a 1:1:1:1 ratio for the compound treatment of pepper seedlings. The formulated compound caused a significant increase in root length, stem thickness, leaf number, dry weight, and chlorophyll content of pepper seedlings. Thus, we obtained a *Bacillus* complex microbial preparation with the potential for growth promotion and biocontrol, which provides improved results for agricultural production while being suitable for use in the natural environment.

Antimicrobial active substances are a class of secondary metabolites produced by both microorganisms and higher plants and animals that are anti-pathogenic and can interfere with other cellular developmental functions. *Bacillus* can secrete three non-ribosomal pathway-synthesized lipopeptide antibiotics, such as surfactin, fengycin, and iturin (Li et al., 2010). López et al. (2009) reported that the mechanism by which surfactin promotes the formation of *Bacillus ubtilis* 3610 biofilms is related to its ability to cause potassium ion leakage in bacterial cell membranes. The analogs of fengycin were isolated and purified from *Bacillus thuringiensis* CMB26, which has antifungal and bacterial activities (Kim et al., 2004). Two congeners of the iturin family, bacillomycin D, were also isolated and purified from the fermentation broth of *Bacillus subtilis* AU195, with a strong inhibitory effect on *Aspergillus flavus* (Moyne et al., 2001). In the time-of-flight mass spectrometry results tested in this study, strains HN-2, FZB42, and HAB-2 containing the C_15–17_ surfactin component all showed significant hemolytic activity against blood plates (**Figure 3A**), and the rate of biofilm formation was faster than that of the other strains, which is consistent with what was previously reported. We suggested that plants treated with these four strains can reduce the colonization of pathogenic bacteria on the host plant because of the presence of surfactin, which in turn reduces the occurrence of the disease. The strains FZB42, HN-2, and HAB-2 contained C_15,18_ fengycin components as determined by time-of-flight mass spectrometry, and these three *Bacillus* strains showed significant antagonistic ability against *Xoo* compared with strains 168 and HAB-5, which did not contain fengycin components (**Figure 3C**). Notably, this was similar to the bacterial inhibitory effect of strain CMB26. In addition, the strains FZB42, HN-2, and HAB-2 containing C_13–18_ iturin components had a significant inhibitory effect on anthracnose *Colletotrichum gloeosporioides* Penz (**Figure 3B**), with an EC_50_ of 38.1 μg/mL. Thus, the antagonistic ability of the lipopeptides against the fungus in the present experiment was better than previously reported. The above-mentioned inhibition experiments combined with the results of time-of-flight mass spectrometry illustrated that strains FZB42, HN-2m, and HAB-2, which contain lipopeptide-active substance components, have biocontrol potential, and are more conducive to the attachment of *Bacillus* to the host plant roots to aid the host plant in adapting to changing environments and to better resist infection by external pathogens.

Bacteria can move across solid surfaces through flagellar-driven swarm motility, allowing movement to more favorable environments (Rütschlin and Böttcher, 2020). Swarm motility promotes bacterial–host plant interactions by enhancing antibiotic resistance and facilitating their movement to the site of infection and attachment to target host cells. Previous work has found that *Bacillus subtilis* OKB105 is a backfill mutant of strain 168 in the *sfp* gene. Therefore, the colony motility of strain FZB42, which contains the lipopeptide surfactin component, is superior to that of strain 168 and its mutant, which is missing the *sfp* gene for lipopeptide synthesis. Tian (2011) showed that *Bacillus subtilis* OKB105 and OKB105*ΔyczE* spread more slowly than strains FZB42 and FZB42*ΔyczE*. In this study, strain FZB42 could swarm to half of the plate, while strain 168 had little swarming ability, which is consistent with previous reports. Here, both strains HN-2 and HAB-2, which also contained surfactin, had significantly better colony motility than strain 168 (**Figure 1**).

Biofilms are multicellular aggregates formed by bacteria bound to solid or liquid media, or other microorganisms in close contact, to adapt to their environment through the adhesion of extracellular matrix (Mielich-Süss and Lopez, 2015). Thus, biofilm formation is a prerequisite for host plant colonization ability and the effectiveness of fungus disease control on host plants (Cai et al., 2019; Liu et al., 2020). Bacterial strains with weak biofilm formation generally result in significantly reduced colonization ability and biocontrol effectiveness (Ma et al., 2012). As an ideal biocontrol microorganism, *Bacillus* can swim rapidly and form biofilms on plant roots, on which microbial rhizosphere colonies beneficial to plant growth are formed and colonize plant roots in large numbers (Wang et al., 2019). Yu (2016) found that *Bacillus subtilis* OKB105 could not form an obvious biofilm, and *Bacillus amyloliquefaciens* FZB42 started to form a thin, flatter layer of the film after 12 h of incubation, which gradually became thicker as the incubation time increased. In contrast, we observed that strain FZB42 and the other four strains of *Bacillus* started to form biofilm after 4 h of incubation, and biofilm could be clearly observed at 12 h when obvious folds appeared. The biofilm showed a more mature three-dimensional structure at 24 h, while strain 168 started to form only biofilm gradually at 24 h. Finally, at 72 h, all six strains formed an obvious biofilm (**Figure 2**), indicating that strain 168 lacked C_15_ surfactin and its biofilm formation ability was significantly lower. Nevertheless, owing to the presence of extracellular polymeric substances, a thin biofilm could still be formed in the late growth stage of the strain, which was consistent with our results from time-of-flight mass spectrometry and was also consistent with Yu’s report. Knockdown of biofilm formation-related genes in different wild-type *Bacillus* species revealed that the rate of film formation in the mutants was greatly reduced and significantly correlated with the ability to colonize (Chen et al., 2012). In this study, strains HN-2 and FZB42 formed biofilms more readily than the other four strains, resulting in stronger inhibition of host plant infestation by the pathogen.

The use of chemical fertilizers to increase yields is an inevitable step in modern agriculture, but it causes many environmental problems. Going forward, the aim of biological agents is to control pests and diseases safely and effectively while also promoting the growth of plants (Erturk et al., 2010; Kumar et al., 2011; Richardson et al., 2009). Kondoh et al. (2001) found that *Bacillus subtilis* RB14 can increase the germination of tomato seeds by 99%. In this study, we tested the effect of six strains of *Bacillus* spp. On the growth of pepper seedlings and found that all six strains could promote the germination of pepper seeds. Notably, the highest germination rate of pepper seeds treated with HN-2 bacterial solution (OD_600_ = 0.9) for 2 h was 100%, which was 15% higher than the control, and therefore preferable to strain RB14 in promoting seed germination. The height of pepper plants inoculated with *Bacillus subtilis* SL-44 increased by 9.00% compared with the control group (Huang, 2018). Volatile organic compounds from *Bacillus subtilis* MT323 could promote the growth of tobacco seedlings with a root length of 23.00 mm, with an observed increase of 48.1% over the control group (Yu et al., 2020). In this study, different absorbances A_600nm_ were observed between strains (OD_600_ of 0.3, 0.5, 0.7, 0.9, and 1.2). When strain HAB-2 (OD_600_ of 0.9) reached 64.44 ± 13.61 mm, the elongation of pepper roots increased at most by 220.12% compared with CK (sterile water) (**Figures 7**–**9**). Therefore, these results indicate that the *Bacillus* in this study is more consistent or even superior in promoting growth than previously reported, and the fermentation solution can produce significant growth-promoting effects on plant seedling germination rate and plant height.

As a growth hormone capable of acting on the whole process of plant growth and development, IAA affects plant cell division, elongation, differentiation, seed germination, root development, and the process of nutritional growth (Gutierrez-Manero et al., 2001; Phillips et al., 2011; Luo et al., 2016). In recent reports, Wu et al. (2018) screened an acid-producing *Klebsiella* strain GMZB-12 with a mean IAA production value of 36.88 mg/L from vegetable garden soil and treated rice seeds by applying this strain GMZB-12, finding that the germination rate and germination potential of rice seeds were increased. In our study, the ability of these six *Bacillus* strains to produce IAA at different times was evaluated, and it was found that the production of IAA in the fermentation broth of strains FZB42, HN-2, HAB-2, and HAB-5 was significantly higher and more stable than that of the other two strains at 36 h. The IAA content in the fermentation broth of strain FZB42 reached 79.46 mg/mL, which was 604.31% of that of strain 168 (**Figure 4**). This is consistent with previous reports, even though the IAA production is higher than that reported by Xu et al.

As an essential element in plants, phosphorus plays an important role in establishing genetic information and energy transportation (Borlotti, 2012). However, most of the phosphorus in the soil exists in forms that are not directly available to plants (Jones et al., 1998). However, *Bacillus* can degrade large molecules into smaller substances for easier absorption. In a study by Liu et al. (2019), a highly efficient phosphorus solubilizing strain (RC01) was screened from safflower inter-rhizosphere soil. RC01 increased phosphorus solubilization with time in Monkina inorganic phosphorus liquid medium, reaching a maximum value of 514.37 mg/L at 120 h when the pH decreased to a minimum. Furthermore, it was found that RC01 causes a continuous decrease in medium pH to reach the maximum phosphorus solubilization during phosphorus solubilization (Liu et al., 2019). In this study, we found that the dissolved insoluble organic phosphorus content in the fermentation broth of strain HAB-5 reached 3.95 mg/mL, which was 131.46% of the dissolved insoluble organic phosphorus content in the fermentation broth of strain 168 (**Figure 7**). This indicates that strains HAB-5, FZB42, HAN-2, and HAB-2 can transform the phosphorus required by the plant and can signal responses under the plant’s own defense metabolic process to induce systemic resistance to pathogens and counteract the effects of an unfavorable plant growth environment on the plant. In conclusion, compared with previous reports, the four strains of *Bacillus* tested in this study have a great ability to promote growth, including higher IAA production and solubilization of insoluble organic phosphorus in the fermentation broth. Furthermore, these strains have more obvious advantages in terms of supplying the elemental ions needed by plants, inducing plants to develop their own resistance to harsh external conditions, and protecting plants from the effects of the disease. However, we found that each *Bacillus* has different growth-promoting effects on plants. Therefore, we selected four strains of *Bacillus* with good growth-promoting effects, which also provided a strong basis for future strain compounding work.

Owing to variable environmental conditions and complex soil systems, the effect of fungicides produced using a single strain is usually less stable (Gu et al., 2018). In addition, a combination of biocontrol fungicides can create an ecological environment that is favorable for the host but not for disease development, which can effectively compensate for the shortcomings of a single strain and improve control efficacy. In recent years, there have also been many reports of using compound broth to promote plant growth. For example, Li et al. (2018) found that a *Bacillus subtilis* B23–1 and *Bacillus amyloliquefaciens* B01–2 combination had a lower incidence than a single agent, and their control effect on wilt disease in *Bambusa pervariabilis × Dendrocalamopsis daii* was 81.5%. Similarly, Chen et al. (2018) combined *Bacillus megaterium* Y-30 and *Bacillus amyloliquefaciens* CM3 in a 2:1 ratio to achieve 59.4 6% bacterial inhibition, which was 25.22% higher than that of the single strain CM3. In this experiment, the individual strains FZB42 (OD_600_ of 1.2), HN-2 (OD_600_ of 0.9), HAB-2 (OD_600_ of 0.9), and HAB-5 (OD_600_ of 1.2) had the greatest effect on pepper growth, and therefore we combined these four strains in a 1:1:1:1 ratio. Importantly, we found that the stem thickness, leaf dry weight, leaf number, and chlorophyll content of pepper seedlings treated with the compound formulation of the bacterial solution increased by approximately 13%, 14%, 26%, and 41%, respectively, compared with the optimal single bacterial solution treatment. Furthermore, and for several of these indicators, pepper seedlings treated with the compound formulation increased by an average of 30% compared with the control water treatment group (**Figures 10**–**13**), which was more beneficial to photosynthesis and the accumulation of organic matter in pepper seedlings. Compared with the previous reports, this study was novel in that it used four kinds of *Bacillus* fermented with a growth-promoting effect for the compound formulation, and the growth-promoting effect was significantly better than the growth-promoting effect of the compound formulation of two kinds of bacteriological agents in previous reports. Compared with the traditional methods of compound formulation of bacteriological agents and chemical pesticides, the formulation in this study contributes more to environmental protection and maintaining balanced ecosystems. Overall, the compound formulation using four strains has the advantage of combining their strengths, providing considerable growth-promoting advantages for each *Bacillus* strain. This results in significant gains in soil inter-root colonization, organic matter accumulation, and photosynthesis of peppers, thus promoting the improvement of pepper yield and quality.

## 5 Conclusion

In summary, we compared the biological characteristics, bacterial inhibition ability, and growth promotion effect of six strains of *Bacillus.* We selected four strains with biocontrol potential and calculated the optimal OD_600_ of these four strains. The strains FZB42 (OD_600_ = 1.2), HN-2 (OD_600_ = 0.9), HAB-2 (OD_600_ = 0.9), and HAB-5 (OD_600_ = 1.2) were compounded in the ratio of 1:1:1:1, and it was found that the growth trend and leaf number of pepper seedlings treated with this compound showed increased growth trends and larger leaf numbers than pepper seedlings treated with the single bacterial solution. This highlights the advantages of the multiple-strain bacterial solution, in addition to the good growth promotion and antagonistic effects on pathogenic bacteria. The use of this biological agent in agricultural systems could reduce the application of chemical pesticides and avoid the imbalances of soil microbial communities, thus reducing the risk of plant disease. This study provides a theoretical basis for the future development and large-scale application of complex bacterial fertilizers using *Bacillus* spp. with different growth-promoting effects to improve the yield and quality of pepper.

## Data availability statement

The original contributions presented in the study are included in the article/supplementary material. Further inquiries can be directed to the corresponding authors.

## Author contributions

YS: performing the experimental work, data curation, writing, reviewing, and editing. HY: experimental design and drafting of the original manuscript. ZL: participated in the experimental work. LC: proposed part of the experimental work. XP: conceptualization and data curation. YW: investigation and methodology. WL: gave advice on experimental design. PJ: presented experimental ideas, designed experiments, guided research, and revised and finalized the article. WM: provided the experimental material preparation and techniques. All authors contributed to the article and approved the submitted version.
